# Protein Kinases: Function, Substrates, and Implication in Diseases

**DOI:** 10.3390/ijms23073560

**Published:** 2022-03-24

**Authors:** Lubos Cipak

**Affiliations:** Department of Genetics, Cancer Research Institute, Biomedical Research Center, Slovak Academy of Sciences, Dubravska Cesta 9, 845 05 Bratislava, Slovakia; lubos.cipak@savba.sk

Protein kinases are important enzymes, involved in the regulation of various cellular processes. Phosphorylation and dephosphorylation provide a rapid and dynamic regulatory mechanism that controls the biological functions of most proteins and act as a molecular switch for diverse regulatory events in signaling pathways that drive cell division, proliferation, metabolism, transcription, differentiation, and apoptosis. From that view, it has become clear that protein kinases play specific regulatory roles throughout the cell, and their defects may result in the development of various diseases.

In this Special Issue “Protein Kinases: Function, Substrates, and Implication in Diseases”, we collected seven review papers and five original research articles, focused on new findings, recent advances and future development in the protein kinase field.

In the first review, DeRoo et al. provides an overview of the current findings supporting a pathologic role of RIPK1 and RIPK3 in cardiovascular disease, and highlights the evidence behind the efficacy of RIPK1 and RIPK3 inhibitors, in the prevention and treatment of cardiovascular disease [1]. The second review, by Janovská et al., introduced the recent findings on the casein kinase 1 enzymes (CK1), their substrates and the therapeutic potential of their inhibition [2]. In the third review, Spinello et al. summarized the general features of CK1α and CK2 protein kinases, presenting the most relevant oncogenic and stress-related signaling nodes regulated by these kinases and report the findings, which support the importance of these two kinases as targets for treatment of hematological cancers [3]. In the fourth review, Jurcik et al. summarized the current advances in the field of chemical genetics in analog-sensitive protein kinases, highlighting strategies for identifying protein kinase substrates and studying the dynamic nature of protein phosphorylation [4]. The fifth review, by Daams and Massoumi, focuses on Nemo-like kinase (NLK), which is an atypical proline-directed serine/threonine mitogen-activated protein (MAP) kinase. The authors present the recent discoveries on this kinase by using NLK-deficient mice, which show a phenotype in the development and function of organs, such as the lung, heart and skeleton [5]. The sixth review, by Kudlik et al., presents the diverse nature of the tyrosine kinase substrates (TKS) scaffold proteins, by discussing their structure, regulation by SRC kinase, relevant signaling pathways, interaction partners, the involvement in various cellular processes and related pathologies [6]. The last review, by Obsilova and Obsil, provides a detailed overview on chaperon protein 14-3-3-mediated kinase regulation, focusing on the most recent mechanistic insights into these important protein–protein interactions, and discussing the structural aspects and functional consequences of such interactions [7].

Concerning the original research articles, Wińska et al. tested the combination effect of 5-fluorouracil (5-FU), with specific inhibitors of protein kinase CK2 in the triple-negative breast cancer cell line. They found that inhibitors of protein kinase CK2 can improve 5-FU-based anticancer therapy and proposed FAK kinase as an attractive target for cancer therapy [8]. In the next research article, Szoltysek et al. focused on DRAK2, a novel stress response kinase that plays a critical role in apoptosis, T-cell biology, and B-cell activation in chronic lymphocytic leukemia (CLL). They found that low expression levels of DRAK2 were significantly associated with unfavorable outcomes in CLL patients. The transcriptome analysis highlighted MAPK, NF-κB and Akt, as critical signaling hubs upon DRAK2 knockdown, thus, indicating DRAK2 as a novel marker of CLL patient survival and prognosis [9]. The third research article, by Sivakova et al., introduced the optimized label-free quantitative (LFQ) phosphoproteomics workflow, based on Fe-IMAC phosphopeptide enrichment, followed by strong anion exchange and porous graphitic carbon fractionation strategies, as a tool to study the dynamic nature of protein phosphorylation [10]. In the fourth research article, Dibus et al. presented data from the screening of substrates of protein kinase N3 (PKN3), a serine/threonine kinase, implicated in the tumor progression of multiple cancer variations. The authors identified a new set of potential PKN3 substrates and revealed a new negative feedback regulatory mechanism of Rho signaling, mediated by PKN3-induced ARHGAP18 activation [11]. The last research article, by Heintze et al., presents the development of diazocine-functionalized derivatives of the VEGFR-2 inhibitor axitinib, as reversibly photoswitchable inhibitors that exhibit a greater than 40-fold difference in biological activities upon irradiation [12].

## Data Availability

Not applicable.

## References

[1] DeRoo E., Zhou T., Liu B. (2020). The Role of RIPK1 and RIPK3 in Cardiovascular Disease. Int. J. Mol. Sci..

[2] Janovská P., Normant E., Miskin H., Bryja V. (2020). Targeting Casein Kinase 1 (CK1) in Hematological Cancers. Int. J. Mol. Sci..

[3] Spinello Z., Fregnani A., Quotti Tubi L., Trentin L., Piazza F., Manni S. (2021). Targeting Protein Kinases in Blood Cancer: Focusing on CK1α and CK2. Int. J. Mol. Sci..

[4] Jurcik J., Sivakova B., Cipakova I., Selicky T., Stupenova E., Jurcik M., Osadska M., Barath P., Cipak L. (2020). Phosphoproteomics Meets Chemical Genetics: Approaches for Global Mapping and Deciphering the Phosphoproteome. Int. J. Mol. Sci..

[5] Daams R., Massoumi R. (2020). Nemo-Like Kinase in Development and Diseases: Insights from Mouse Studies. Int. J. Mol. Sci..

[6] Kudlik G., Takács T., Radnai L., Kurilla A., Szeder B., Koprivanacz K., Merő B.L., Buday L., Vas V. (2020). Advances in Understanding TKS4 and TKS5: Molecular Scaffolds Regulating Cellular Processes from Podosome and Invadopodium Formation to Differentiation and Tissue Homeostasis. Int. J. Mol. Sci..

[7] Obsilova V., Obsil T. (2020). The 14-3-3 Proteins as Important Allosteric Regulators of Protein Kinases. Int. J. Mol. Sci..

[8] Wińska P., Karatsai O., Staniszewska M., Koronkiewicz M., Chojnacki K., Rędowicz M.J. (2020). Synergistic Interactions of 5-Fluorouracil with Inhibitors of Protein Kinase CK2 Correlate with p38 MAPK Activation and FAK Inhibition in the Triple-Negative Breast Cancer Cell Line. Int. J. Mol. Sci..

[9] Szoltysek K., Ciardullo C., Zhou P., Walaszczyk A., Willmore E., Rand V., Marshall S., Hall A., Harrison C.J., Eswaran J. (2020). DAP Kinase-Related Apoptosis-Inducing Protein Kinase 2 (DRAK2) Is a Key Regulator and Molecular Marker in Chronic Lymphocytic Leukemia. Int. J. Mol. Sci..

[10] Sivakova B., Jurcik J., Lukacova V., Selicky T., Cipakova I., Barath P., Cipak L. (2021). Label-Free Quantitative Phosphoproteomics of the Fission Yeast Schizosaccharomyces pombe Using Strong Anion Exchange- and Porous Graphitic Carbon-Based Fractionation Strategies. Int. J. Mol. Sci..

[11] Dibus M., Brábek J., Rösel D. (2020). A Screen for PKN3 Substrates Reveals an Activating Phosphorylation of ARHGAP18. Int. J. Mol. Sci..

[12] Heintze L., Schmidt D., Rodat T., Witt L., Ewert J., Kriegs M., Herges R., Peifer C. (2020). Photoswitchable Azo- and Diazocine-Functionalized Derivatives of the VEGFR-2 Inhibitor Axitinib. Int. J. Mol. Sci..

